# Obsessive–compulsive symptoms and overall psychopathology in psychotic disorders: longitudinal assessment of patients and siblings

**DOI:** 10.1007/s00406-016-0751-0

**Published:** 2016-12-17

**Authors:** Frederike Schirmbeck, Marije Swets, Carin J. Meijer, Mathias Zink, Lieuwe de Haan, GROUP investigators, René S. Kahn, Jim van Os, Richard Bruggeman, Wiepke Cahn, Agna A. Bartels-Velthuis, Inez Myin-Germeys

**Affiliations:** 1 Department of Psychiatry, Academic Medical Centre, University of Amsterdam, Amsterdam, The Netherlands; 2 Arkin Institute for Mental Health, Amsterdam, The Netherlands; 3 Central Institute of Mental Health, Medical Faculty Mannheim, University of Heidelberg, Mannheim, Germany

**Keywords:** Psychosis, Schizophrenia, Obsessive–compulsive, Comorbidity, Longitudinal

## Abstract

The course of obsessive–compulsive symptoms (OCS) and its association with alterations in other clinical variables in patients with psychotic disorders is insufficiently known. Patients (*n* = 602) and unaffected siblings (*n* = 652) from the Dutch Genetic Risk and Outcome of Psychosis (GROUP) study were investigated at baseline and after 3 years. Participants were assigned to four groups based on the course of OCS over time: no-OCS, persistent OCS, initial OCS and de novo OCS. In addition to cross-sectional comparisons, longitudinal associations between changes in OCS and symptoms of schizophrenia and general functioning were investigated. Patients with co-occurring OCS reported significantly higher severity of psychotic and affective symptoms as well as lower overall functioning compared to patients without OCS. These differences were stable over time for patients reporting persistent OCS. Subsequent repeated measure analysis revealed significant interaction effects for groups reporting changes in their OCS. Whereas the group with remission of initial OCS showed significant improvement in positive symptoms, emotional distress and functioning, the de novo group showed no significant change in these variables, but rather reported stable higher psychopathology. Similar results were found on a subclinical level in siblings. Patients with co-occurring OCS present a more severe clinical picture, especially if symptoms persist over time. The remission of OCS was associated with overall improvement, whereas individuals with de novo OCS already reported higher clinical impairment before OCS onset. More research is needed to elucidate causal pathways and to develop effective interventions for persistent comorbid OCS.

**Electronic supplementary material:**

The online version of this article (doi:10.1007/s00406-016-0751-0) contains supplementary material, which is available to authorized users.

## Introduction

Recent meta-analyses conclude that 12% of patients with schizophrenia also fulfil the criteria for obsessive–compulsive disorder (OCD) and that about 30% report obsessive, distressing, intrusive thoughts and related compulsions [1–3]. A recent longitudinal cohort study in Sweden showed that individuals with OCD had an increased risk of late diagnosis of schizophrenia and vice versa [4]. Hence, the co-occurrence of OCD and psychotic disorders is a common problem, which has led to an increased research interest over the last decade.

The question how co-occurring obsessive–compulsive symptoms (OCS) interact with other clinical characteristics in schizophrenia patients led to conflicting findings. Early concepts hypothesized that patients with schizophrenia develop OCS in an attempt to reduce psychotic symptoms, and thus, the presence of OCS was thought to have a protective effect regarding psychotic disintegration [5, 6]. Subsequent research which investigated the clinical effect of comorbid OCS in schizophrenia showed greater levels of hopelessness [7], lower quality of life [8], more social dysfunction [9, 10] and a higher degree of cognitive impairment [11, 12]. Consequently, patients with schizophrenia and comorbid OCS were reported to have a less favourable prognosis [13–17]. Regarding the relationship between OCS and severity of schizophrenia symptoms, findings yielded inconclusive and to some degree contradictory results [18]. A meta-analysis by Cunill et al. [14] concluded that most studies reported more severe positive, and negative symptoms if OCS were present. However, several studies did not find significant associations [10, 19–21], and others even reported lower severity of negative symptoms [22] or lower levels of positive and negative symptoms in patients with a first psychotic episode and comorbid OCS [23, 24]. A finding that has consistently been replicated is the association between comorbid OCS and more severe depressive symptoms [10, 24–27].

So far, research assessing the association between co-occurring OCS, affective and psychotic symptoms has mainly been investigated in cross-sectional designs. Group comparisons are therefore often restricted to the presence or absence of OCS at one single assessment time. Fontenelle et al. [28] investigated OCD in a cohort of individuals at ultra-high risk of psychosis and found especially persistent OCD related to the development of a psychotic disorder 7 years later. Another recent prospective study investigated the 5-year course of OCS in a sample of first-episode patients. de Haan et al. [10] found no differences in positive and negative symptoms between patients with versus without OCS at any of the three assessments, nor did the presence of initial OCS predict time to relapse or symptomatic recovery 5 years later. To the best of our knowledge, so far no study has investigated the course of OCS and associated changes in the severity of schizophrenia symptoms over time. Furthermore, nothing is known about the course of OCS and its relation with subclinical psychotic and depressive symptoms in relatives of patients with a psychotic disorder. Research in unaffected family members offers a unique possibility to study associations between changes in OCS and alterations in subclinical symptoms without confounding therapy-related factors.

The aim of the present study was to investigate the longitudinal course of co-occurring OCS and its relationship with psychotic symptoms, affective symptoms and social functioning in a large cohort of patients with psychotic disorders. In addition, associations between OCS and subclinical symptoms in a cohort of unaffected siblings were investigated as a unique validation approach. Based on the above-reported literature, we hypothesize that persistent comorbid OCS are related to more severe symptoms of schizophrenia and depression and to more severe functional impairment. We further propose that the de novo development of OCS is associated with an increased severity of these clinical variables, whereas remission of initial OCS is associated with a decreased severity.

## Methods

### Study design and participants

The study sample was part of the multicenter study ‘Genetic Risk and Outcome of Psychosis’ (GROUP). Baseline and 3-year follow-up assessments of patients and siblings with complete data sets were included in the study. The procedure of recruitment and population characteristics have been described in detail elsewhere [29]. In short, inclusion criteria for patients and siblings were (1) age range of 16–50 years and (2) good command of the Dutch language. Patients had to meet DSM-IV-TR criteria for a non-affective psychotic disorder [30] which was assessed with the Comprehensive Assessment of Symptoms and History (CASH [31]) or the Schedules for Clinical Assessment for Neuropsychiatry version 2.1 (SCAN [32]). An additional inclusion criterion for the sibling group was the absence of a lifetime psychotic disorder. All participants provided written informed consent prior to their inclusion in the study, which was approved by the accredited Medical Ethics Review Committee (METC).

### Clinical measures

Sociodemographic data on age, gender, education level, age of onset, duration of illness and medical treatment were collected. Current diagnosis of cannabis abuse and dependence was assessed using the Composite International Diagnostic Interview (CIDI [33]). Severity of OCS was measured with the Yale–Brown Obsessive–Compulsive Scale (YBOCS [34]), which has been validated for the assessment of OCS in schizophrenia [35, 36]. Severity of positive and negative symptoms and emotional distress in patients was assessed with the Positive and Negative Syndrome Scale (PANSS [37]) according to the five-factor model (positive subscale: P1, P3, P5, P6, G9; negative subscale: N1, N2, N3, N4, N6, G7, G8, G16; and emotional distress: G2, G3, G4, G6) [38]. The Community Assessment of Psychic Experiences (CAPE [39]) is a 42-item self-report questionnaire, which was used to assess frequency and associated distress with mild psychotic experiences in siblings. Three subscales cover positive psychotic experiences, negative psychotic experiences and depressive feelings. In the current study, only frequency ratings were included as outcome measure. Social functioning was measured using the Global Assessment of Functioning Scale (GAF [40]) with separate ratings for symptoms and degree of disability [41]. Before the start of the study, all interviewers received extensive training workshops to practise the assessments of all measures used in the GROUP project (for details see [29]).

### Statistical analysis

Statistical analyses were performed using the Statistical Package for Social Sciences (SPSS version 18.0, Chicago, IL, US). Sociodemographic and psychopathological characteristics were compared between the groups using multivariate analysis of variance (MANOVA) and *χ* ^2^ tests.

Fixed-effect regression models were used for PANSS/CAPE and GAF outcome measures with the different OCS groups (no-OCS, persistent, remission or de novo) as the fixed part of the model and covariates to account for significant between-group differences in medical history in patients. These models were separately analysed for the two status groups (patients and unaffected siblings) at baseline and follow-up. Bonferroni corrections for multiple comparisons were applied for subsequent pairwise analyses.

To assess longitudinal changes in clinical characteristics over time, we calculated repeated measure analysis of variances (rmANOVAs) and post hoc paired *t* test with the OCS group as the fixed part of the model and psychopathology and functioning as the dependent variables. These models were analysed separately within the factor stability (no changes vs. changes in reported OCS) and the two status groups (patients and siblings). Effect sizes were calculated to complement statistical significance of within-group changes (Cohen’s *dz*) and to give an estimation of small (*dz* = 0.2), medium (*dz* = 0.5) or large (*dz* = 0.8) effects. Correlation analyses assessed the association between changes in the severity of OCS and other clinical variables.

## Results

### Sociodemographic characteristics and clinical assessments

We included 602 patients and 652 siblings in our analyses with complete data of sociodemography, YBOCS and PANSS/CAPE scores for both assessments. Based on interpretation guidelines, we defined the presence of OCS as a YBOCS total score of at least 8, representing mild symptom severity [34]. 31.0% of patients and 7.8% of siblings fulfilled this criterion at least at one assessment. The majority of participants reported changes in their comorbid OCS between assessments either as newly occurring de novo OCS (12.0%) or as remission of initially reported OCS (11.6%); only 7.5% reported persistent OCS over time. Therefore, we created the following groups within the patient sample: OCS de novo (*n* = 72): development of at least mild OCS at follow-up; OCS remission (*n* = 70): remission of initially reported OCS at follow-up; persistent OCS (*n* = 45): at least mild OCS at both assessment times; and no-OCS group (*n* = 415): no relevant OCS at either time point. Accordingly, unaffected siblings were assigned to a de novo OCS group (*n* = 25), OCS remission group (*n* = 26) or no-OCS group (601). Because only five siblings presented persistent OCS, we did not include this group in our main analyses.

Table 1 presents between-group comparisons on demographic and clinical variables. The four patient groups did not significantly differ in age, gender, ethnicity, education, diagnosis, current cannabis abuse/dependence or estimated total IQ. Patients in the remission group showed a trend towards an earlier age at onset when compared to the no-OCS group [mean difference (md = 2.1446, *p* = .054)], and the persistent group reported longer illness duration compared to the no-OCS group (md = 1.643, *p* = .028). Comparisons within siblings did not reveal any significant group differences (Table 1). According to group definition, baseline and follow-up assessment of OCS severity differed between the above-defined groups (Table 1).

**Table 1:** Sociodemographic and clinical characteristics in patients and siblings at baseline

Patients	OCS de novo	OCS remission	Persistent OCS	No-OCS	Between-group differences
	*N* = 72	*N* = 70	*N* = 45	*N* = 415	
Age	26.9 ± 5.8	25.6 ± 6.5	27.2 ± 6.0	27.4 ± 7.4	*F*_3,598_ = 1.380, *p* = .248
Gender (male/female)	58/14	55/15	36/9	319/96	*χ* ^2^ = 0.673, *p* = .879
Ethnicity (Caucasian/others)	58/14	57/13	35/10	347/68	*χ* ^2^ = 1.298, *p* = .730
Age of onset	21.9 ± 5.4	21.2 ± 6.1	21.4 ± 6.2	23.3 ± 6.5	*F*_3,598_ = 3.709, *p* = .012
Duration of illness	4.9 ± 3.8	4.4 ± 3.3	5.8 ± 4.2	4.2 ± 3.7	*F*_3,598_ = 3.289, p = .020
Education in years	13.0 ± 3.6	12.8 ± 4.0	12.3 ± 4.8	12.6 ± 3.7	*F*_3,581_ = 0.352, p = .788
WAIS estimated total IQ	93.8 ± 14.2	95.7 ± 15.2	94.7 ± 15.9	97.3 ± 16.5	*F*_3,574_ = 1.097, *p* = .350
Cannabis abuse/dependence (yes/no)	16/56	17/53	4/41	76/339	*χ* ^2^ = 4.898, *p* = .179
Diagnosis					
Schizophrenia	53	48	35	259	*χ* ^2^ = 16.727, *p* = .335
Schizophreniform disorder	1	4	2	34	
Schizoaffective disorder	10	11	6	61	
Delusion disorder	1	0	0	11	
Brief psychotic episode	0	0	1	10	
Psychotic disorder NOS	7	7	1	40	
YBOCS					
Obsessions	0.5 ± 1.4	6.7 ± 4.6	6.5 ± 5.2	0.1 ± 0.7	
Compulsions	0.3 ± 1.1	6.1 ± 4.8	7.4 ± 4.6	0.1 ± 0.5	
Total	0.8 ± 1.7	12.8 ± 5.6	13.8 ± 6.0	0.2 ± 0.8	

Siblings	OCS de novo	OCS remission	No-OCS	Between-group differences
	*N* = 25	*N* = 26	*N* = 601	
Age	28.2 ± 8.4	27.6 ± 9.0	27.8 ± 8.0	*F*_2,649_ = 0.036, *p* = .965
Gender (male/female)	8/17	8/18	276/325	*χ* ^2^ = 3.993, p = .136
Ethnicity (Caucasian/others)	23/2	24/2	522/79	*χ* ^2^ = 1.182, *p* = .554
Education in years	14.8 ± 3.6	14.5 ± 4.6	13.8 ± 3.7	*F*_2,637_ = 1.360, *p* = .258
WAIS estimated total IQ	99.0 ± 17.5	102.8 ± 15.8	104.5 ± 15.6	*F*_2,637_ = 1.517, *p* = .220
Cannabis abuse/dependence (yes/no)	1/24	3/23	28/573	*χ* ^2^ = 2.786, *p* = .426
YBOCS				
Obsessions	0.0 ± 0.2	3.7 ± 4.3	0.0 ± 0.2	
Compulsions	0.4 ± 1.4	6.1 ± 3.4	0.1 ± 0.6	
Total	0.5 ± 1.4	9.8 ± 4.9	0.1 ± 0.7	

*OCS* obsessive–compulsive symptoms, *WAIS* Wechsler Adult Intelligence Scale, *YBOCS* Yale–Brown Obsessive–Compulsive Scale

Regarding current intake of medication, data on current use of antipsychotic and antidepressive medication were available in a subsample of 369 patients. The number of patients treated with clozapine compared to all other antipsychotic medication did not significantly differ at baseline, but reached significance at follow-up (*χ* ^2^ = 10.264, *p* = .016). *Post hoc* comparisons revealed that significantly more patients in the de novo (*χ* ^2^ = 4.167, *p* = .041) and persistent (*χ* ^2^ = 7.782, *p* = .005) group received CLZ compared to the no-OCS group.

Comparisons on the frequency of antidepressant treatment revealed significant between-group differences at follow-up (*χ* ^2^ = 22.582, *p* < .001). *Post hoc* pairwise comparisons showed that the de novo group and persistent group were more frequently treated with antidepressants than the no-OCS group (*χ* ^2^ = 10.213, *p* = .001; *χ* ^2^ = 17.623, *p* < .001) and patients in the persistent group were also more likely to be treated with antidepressants than in the remission group (*χ* ^2^ = 4.271, *p* = .039).

Although numbers were small, in an explorative approach, we assessed whether the risk for siblings to present with OCS at baseline or follow-up was greater for those siblings of patients, who report OCS at least once during the assessment period. Relative risk calculations were done in a subsample of 467 patient–sibling pairs from the same families. Compared to siblings of patients of the no-OCS group, siblings of patients of the initial persistent or de novo group did not show significantly increased relative risks to report OCS at baseline or follow-up. For more details, see Table 1 in supplementary material.

### Cross-sectional between-group differences at baseline and follow-up

To assess between-group differences at baseline and follow-up in patients, multivariate analyses of variance were calculated. To account for differences in age at onset and illness duration, these variables were added as covariates to the models. Analyses revealed significant results for all PANSS and GAF scores at both assessments except for a non-significant trend for the PANSS negative subscale at follow-up (Table 2). *Post hoc* pairwise analyses showed significantly higher PANSS positive and emotional distress scores, as well as lower GAF symptom scores for the three groups with persistent (md = 4.16, *p* < .001; md = 4.46, *p* < .001; md = 7.32, *p* = .023), remission (md = 2.92, *p* = .001, md = 3.58, *p* = <.001; md = 7.56, *p* = .002) and de novo OCS (md = 2.44, *p* = 002; md = 2.41, *p* = .003; md = 4.18, *p* = .046) when compared to the no-OCS group at baseline. The persistent group also showed significantly higher PANSS negative and lower GAF disability scores compared to the no-OCS group (md = 3.36, *p* = .004; md = 9.09, *p* = .001).

**Table 2:** Between-group differences in PANSS and GAF scores at baseline and 3-year follow-up in patients

	OCS de novo	OCS remission	Persistent OCS	No-OCS	Between-group differences
	*N* = 72	*N* = 70	*N* = 45	*N* = 415	MANCOVA
*Baseline*					
PANSS					
Positive scale	14.6 ± 6.4	15.6 ± 6.4	16.8 ± 6.7	12.2 ± 5.9	*F*_3,594_ = 11.647, *p* < .001
Negative scale	15.0 ± 6.0	15.0 ± 6.6	17.1 ± 6.1	14.1 ± 6.3	*F*_3,594_ = 4.450, *p* = .004
Emotional distress	17.0 ± 5.3	18.1 ± 5.8	19.0 ± 5.9	14.3 ± 5.2	*F*_3,594_ = 18.523, *p* < .001
GAF					
Symptoms	54.4 ± 15.5	50.2 ± 17.4	51.4 ± 15.4	58.6 ± 15.6	*F*_3,576_ = 7.537, *p* < .001
Disability	55.4 ± 14.7	52.7 ± 15.3	48.6 ± 14.2	57.2 ± 15.5	*F*_3,576_ = 5.709, *p* = .001
*Follow*-*up*					
PANSS					
Positive scale	14.6 ± 6.0	12.0 ± 6.4	15.9 ± 6.4	10.9 ± 5.3	*F*_3,595_ = 14.746, p < .001
Negative scale	13.2 ± 4.8	12.2 ± 4.6	14.6 ± 6.5	12.4 ± 5.6	*F*_3,595_ = 2.603, *p* = .051
Emotional distress	16.2 ± 4.6	14.5 ± 5.4	17.9 ± 6.1	12.5 ± 4.6	*F*_3,595_ = 24.363, *p* < .001
GAF					
Symptoms	52.8 ± 16.1	59.2 ± 16.3	51.1 ± 13.1	62.0 ± 15.8	*F*_3,562_ = 11.518, *p* < .001
Disability	54.2 ± 14.7	61.7 ± 14.5	50.0 ± 11.9	62.9 ± 16.1	*F*_3,562_ = 13.912, *p* < .001

*PANSS* Positive and Negative Syndrome Scale, *GAF* General Assessment of Functioning, *OCS* obsessive–compulsive symptoms

Three years later, the persistent group still showed significantly more impairment compared to the no-OCS group (PANSS positive: md = 4.58, *p* < .001; emotional distress: md = 5.21, *p* < .001; GAF symptoms: md = 11.12, *p* = <.001; and GAF distress: md = 13.29, *p* < .001) and higher PANSS positive and lower GAF disability scores (md = 3.56, *p* = .006; md = 11.30, *p* = 001) compared to the remission group. Accordingly, the de novo group reported significantly higher scores compared to the no-OCS group on measures of positive symptoms (md = 3.54, *p* < .001), emotional distress (3.63, *p* = <.001), lower GAF symptom (md = 9.16, *p* < .001) and disability scores (md = 8.88, *p* < .001). Furthermore, the de novo group scored higher on the PANSS positive subscale (md = 2.52, *p* = .046) and lower on the GAF disability subscale (md = 6.90, *p* = .021) compared to the remission group. All significant group differences between the remission and no-OCS group diminished except for a remaining higher score on the emotional distress subscale (md = 2.00, *p* = .009).

Comparable analyses in siblings revealed significant results for all CAPE subscales at baseline and follow-up (Table 3). *Post hoc* analyses at baseline showed significantly higher scores of the OCS remission and de novo group on CAPE positive (md: 4.63, *p* < .001; md: 2.22, *p* = .015), negative (md: 5.37, *p* < .001; md: 2.09, *p* = .004) and depressive symptoms (md: 3.36, *p* < .001; md: 1.62, *p* = .009) when compared to the no-OCS. The remission group also scored higher on all three outcome variables when compared to the de novo OCS group (md: 2.40, *p* = .05; md: 1.74, *p* = .040; md: 3.27, *p* = .022). Three years later, the remission and de novo groups still scored significantly higher on all CAPE subscales compared to the no-OCS group (positive: md: 3.29, *p* < .001; md: 3.63, *p* < .001; negative: md: 5.12, *p* < .001; md: 5.34, *p* < .001; depressive: md: 3.10, *p* < .001; md: 2.75, *p* < .001), but all differences between the remission and de novo group diminished.

**Table 3:** Between-group differences in CAPE scores at baseline and 3-year follow-up in siblings

	OCS de novo	OCS remission	No-OCS	Between-group differences
	*N* = 25	*N* = 26	*N* = 601	MANOVA
*Baseline*				
CAPE				
Positive	6.4 ± 4.7	8.8 ± 9.4	4.1 ± 4.1	*F*_2,649_ = 15.800, *p* < .001
Negative	9.8 ± 4.7	13.1 ± 7.8	7.7 ± 5.0	*F*_2,649_ = 15.476, *p* < .001
Depressive	6.7 ± 3.1	8.4 ± 5.5	5.1 ± 2.9	*F*_2,649_ = 18.282, *p* < .001
*Follow*-*up*				
CAPE				
Positive	5.8 ± 9.8	5.5 ± 8.1	2.2 ± 3.2	*F*_2,649_ = 17.981, *p* < .001
Negative	11.7 ± 7.2	11.5 ± 8.3	6.4 ± 5.6	*F*_2,649_ = 19.345, *p* < .001
Depressive	6.6 ± 4.2	7.0 ± 5.2	3.9 ± 3.1	*F*_2,649_ = 19.030, *p* < .001

*CAPE* Community Assessment of Psychic Experiences, *OCS* obsessive–compulsive symptoms

### The longitudinal course of OCS and associated changes in psychopathology and general functioning

To analyse the course of symptom severity and the impact of change versus stability in OCS over time, we subsequently calculated separate repeated measure ANOVAs for the de novo group versus remission group and persistent group versus no-OCS group within the patient sample.

Comparisons between the de novo group and the remission group showed significant group x time interaction effects for the PANSS positive (*F*_1,140_ = 9.986, *p* = .002) and emotional distress scores (*F*_1,140_ = 8.323, *p* = .005), as well as for GAF symptoms (*F*_1,129_ = 9.915, *p* = .002) and disabilities (*F*_1,129_ = 11.850, *p* = .001).

Subsequent group-specific post hoc paired *t* tests showed significant symptom reduction over time within the remission group for the positive (*T*_69_ = 4.533, *p* < .001, *dz* = −.54), negative (*T*_69_ = 3.390, *p* = .001, *dz* = −.41) and emotional distress (*T*_69_ = 4.805, *p* < .001, *dz* = −.33) subscale of the PANSS. Analyses further revealed significant improvement in GAF scores (symptoms: *T*_63_ = −3.299, *p* = .002, *dz* = −.41; disability: *T*_63_ = −3.745, *p* < .001, *dz* = −.47).

**Fig. 1:**
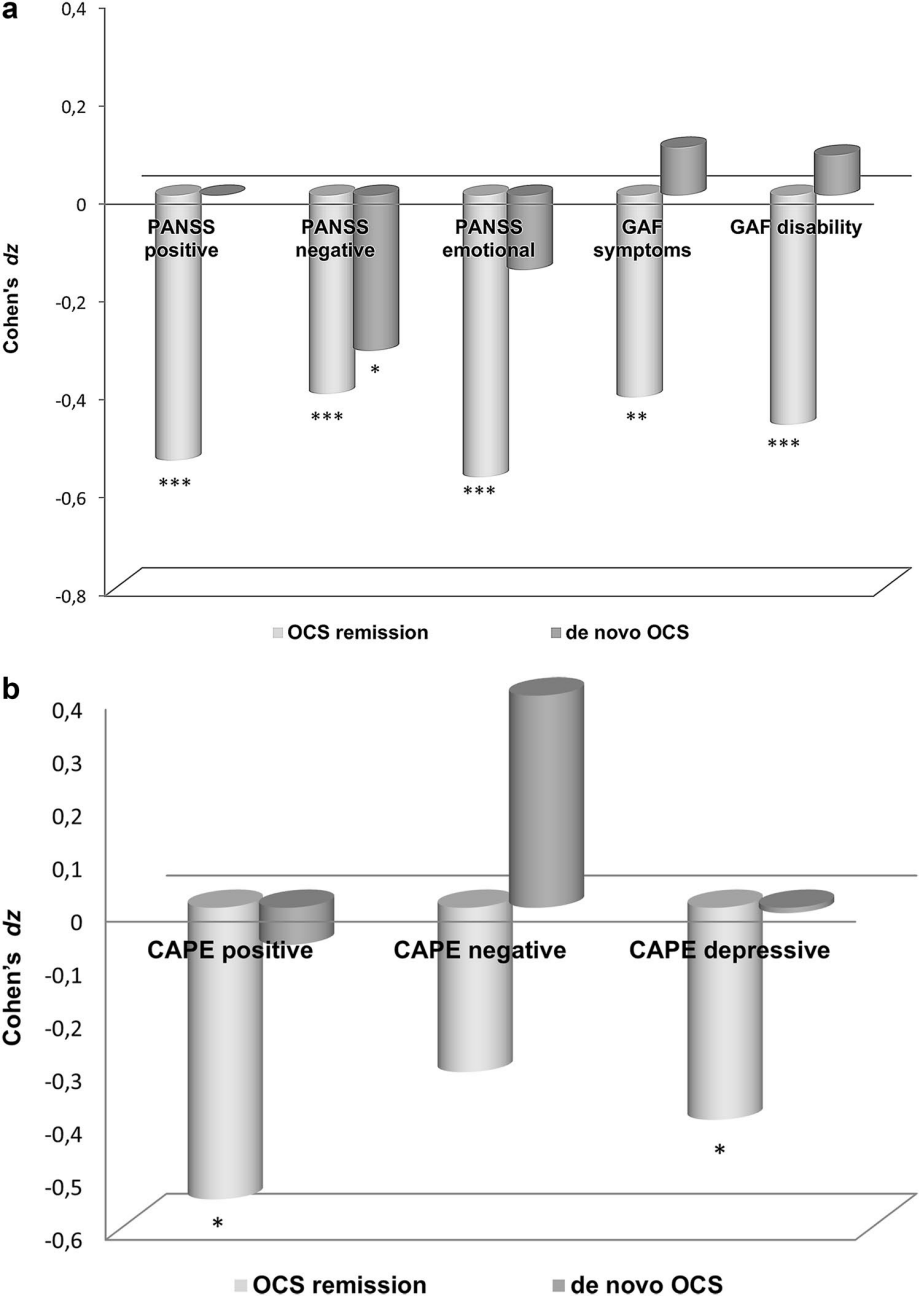
Cohen’s *dz* effect sizes for within-group changes in patients and siblings. The figures show standardized mean differences for within-group changes over time referred to as Cohen’s *dz*. 0 indicates no change in outcome measures between baseline and follow-up; − indicates decrease in symptoms/distress; + indicates increase in symptoms/distress. *p < 0.05, **p < 0.005, ***p < 0.001

Within the de novo group post hoc paired *t* tests showed no significant changes over time, except for significant symptom reductions in the PANSS negative subscale (*T*_71_ = 2.683, *p* = .009, *dz* = −.32). The within-group changes in patients are shown in Fig. 1a.

On a dimensional level changes in YBOCS severity correlated significantly with changes in the PANSS positive subscale (*r* = .22, *p* = .009), the PANSS emotional distress scale (*r* = .20, *p* = .018) and the two GAF subscales symptoms (*r* = −.24, *p* = .006) and distress (*r* = −.25, *p* = .004).

Equivalent analyses were performed within the sample of unaffected siblings. The repeated measure ANOVA comparing participants with OCS in remission to those with de novo occurrence showed one significant group × time interaction effect for the CAPE depressive scale (*F*_1,49_ = 6.375, *p* = .015) and a significant time effect for the positive subscale (*F*_1,49_ = 4.321, *p* = .043). *Post hoc* paired *t* tests revealed significant reduction in subclinical positive symptoms (*T*_25_ = 2.805, *p* = .010, *dz* = −.55) and depressive symptoms (*T*_25_ = 2.060, *p* = .050, *dz* = −.40) within the OCS-remission group. Paired analyses within the de novo group again showed no significant changes in these variables, but a trend for increased CAPE negative symptoms (*T*_24_ = −2.018, *p* = .055, *dz* = .40). The within-group changes in siblings are shown in Fig. 1b. Regarding correlations between change scores, we found significant associations between changes in YBOCS severity and changes in all three CAPE subscales (positive: *r* = .37, *p* = .008; negative: *r* = .33, *p* = .017; depressive: *r* = .30, *p* = .030).

In addition to the comparisons between groups, which showed changes in reported OCS, we analysed between-group differences in patients reporting either persistent OCS or no-OCS. Repeated measure ANOVAs, with illness duration as covariate, showed significant group effects for all three PANSS subscales (positive: *F*_1,453_ = 34.167, *p* < .001; negative: *F*_1,453_ = 12.252, *p* = .001; emotional distress: *F*_1,453_ = 53.511, *p* < .001) and time effects for the PANSS negative (*F*_1,453_ = 20.830, *p* < .001) and emotional distress subscale (*F*_1,453_ = 7.345, *p* = .007). No significant interaction effects were observed. Analyses of the two GAF scales also revealed significant group effects (symptoms: *F*_1,424_ = 18.887, *p* < .001; disability: *F*_1,424_ = 24.814, *p* < .001) and a significant time effect for the disability subscale (*F*_1,424_ = 13.079, *p* < .001). Subsequent post hoc paired *t* tests showed significant improvement in all reported variables within the no-OCS group (positive: *T*_425_ = 4.450, *p* < .001, *dz* = −.22, negative: *T*_425_ = 5.532, *p* < .001, *dz* = −.26, emotional distress: *T*_425_ = 6.404, *p* < .001, *dz* = −.33, GAF symptoms: *T*_384_ = −3.484, *p* = .001, *dz* = −.19, and GAF disability: *T*_384_ = −5.964, *p* < .001, *dz* = −.34), whereas we did not observe any significant changes within the persistent OCS group, except for a significant reduction in the PANSS negative subscale (*T*_44_ = 2.801, *p* = .008, *dz* = −.38).

## Discussion

A fair number of studies assessed the association between co-occurring OCS, psychotic symptoms and social functioning in patients with schizophrenia. However, results have been inconsistent and until now little is known about the longitudinal course and interaction of these variables [18]. In accordance with a prospective study of the 5-year course of comorbid OCS in a cohort of first-episode patients [10], only a minority of our patients reported persistent OCS and the majority reported variation in course as either remission or de novo occurrence of OCS in 3 years.

In line with our first hypothesis, the presence of comorbid OCS was associated with higher severity in schizophrenia-related symptom clusters and with more impairment in social and vocational functioning. Patients, who reported OCS at least once during the assessment period, reported more positive symptoms and emotional distress as well as lower overall functioning when OCS were present, compared to the no-OCS group. In addition, a significant group effect in the repeated measure analyses showed that these associations remained stable over time in patients suffering from persistent comorbid OCS. Interestingly, we found similar results within the groups of unaffected siblings. Siblings showed higher scores in subclinical positive and depressive symptoms when experiencing OCS compared to those who either never reported OCS or did not experience OCS at the time of assessment.

Regarding the hypothesized association between changes in OCS and changes in severity of other symptoms, repeated measure analyses and post hoc comparisons revealed that the remission group in patients showed significant decrease in positive and negative symptoms and emotional distress and improvement in the level of functioning over time. However, contrary to expectations, the de novo group did not report significant increase in these variables over time, but rather already reported higher scores in positive symptoms and emotional distress at baseline compared to the no-OCS group. Similar results were found in siblings, with the remission group showing significant reduction in subclinical positive and depressive symptoms. Siblings in the de novo group showed a trend for an increase in negative symptoms, but again already reported more subclinical positive, negative and depressive symptoms prior to the presence of OCS.

Our results suggest a more severe clinical picture for individuals suffering from both schizophrenia and OCS, supporting previous findings, which consistently reported more depressive symptoms and more impairment in social and vocational functioning [10, 18, 24]. The increased severity of emotional distress when OCS were present is not surprising given that depressive symptoms have been found associated with all co-occurring anxiety disorders in psychotic disorders [42] and the fact that depression is the most common comorbid disorder in OCD individuals [43]. Noteworthy, higher severity of emotional distress was already present at baseline in patients and siblings reporting de novo OCS 3 years later. Rickelt et al. [44] recently investigated the reciprocal influence between obsessive–compulsive and depressive symptoms in OCD patients and found that depressive symptoms at baseline predicted OCS severity 1 year later. According to their hypothesis, rumination, worries and doubt, which frequently go along with emotional distress and are strongly related to obsessive thoughts, might be associated with a subsequent attempt to reduce resulting anxiety through compulsive behaviour.

Furthermore, analyses revealed that the presence of OCS was consistently associated with more positive symptoms in patients and on a subclinical level in siblings. These results stand in line with the meta-analytic conclusion by Cunill et al. [14]. However, results contradict earlier studies, which did not find an association, including the repeated investigation of a large first-episode sample [10]. In line with earlier assumptions, we suggest that the heterogeneity in findings might result not only from differences in study designs, but also from differences in sample characteristics regarding symptom severity and illness duration [14]. Our findings stress the importance to account for changes in symptom clusters, because in line with the study by de Haan et al. [10] initial OCS were not necessarily associated with more psychotic symptoms in the future. Regarding the association with negative symptoms, findings were inconclusive. We only found significantly higher severity of negative symptoms in the patient group reporting persistent OCS at baseline, but these decreased over time. Similarly, negative symptoms slightly decreased in the de novo patients group, but increased on a subclinical level in siblings. Further subgroup analyses might elucidate possible interrelations between OCS and negative symptoms. Lysaker, for example, only found more negative symptoms in a subsample of comorbid OCS patients with poorer functioning and more cognitive impairment [15].

The more consistent link between comorbid OCS and positive symptoms in our sample raises the question of causality. It has been suggested that the association between co-occurring OCS and greater severity of psychotic symptoms could simply be an artefact of the phenomenological overlap of the two disorders [14]. Distinguishing obsessions and compulsions from delusions and hallucinations is indeed a diagnostic challenge, if patients show poor insight into their obsessions or display compulsions that resemble stereotypic mannerisms [45, 46]. However, assuming symptom overlap, we would expect simultaneous changes within our alternating groups, but only observed a decrease in psychotic symptoms in the remission group and no changes in the de novo group. Furthermore, the reliably identification of comorbid OCS with the YBOCS in patients with schizophrenia has been shown [35, 36]. It is therefore unlikely that diagnostic artefacts explain our findings and the significant co-occurrence observed between the two disorders in general.

So far, attempts to elucidate possible pathophysiological mechanisms of the co-occurrence of OCD and schizophrenia have been inconclusive and suggest multicausal pathways for different subgroups [47]. Increasing evidence suggests a shared genetic vulnerability based on familial aggregation of schizophrenia–spectrum disorders and OCD [4, 48]. Our exploratory analyses do not support the notion of an increased relative risk for siblings to experience OCS if their sibling(patient) belongs to one of the OCS groups. However, these analyses are limited by small sample sizes resulting in large confidence intervals and have to be interpreted with great cautious. Furthermore, common neurobiological mechanisms have been proposed to underlie both obsessive–compulsive and psychotic disorders and may explain the high percentage of their co-occurrence [49, 50]. Neuroimaging studies describe abnormalities of the frontal lobe, the basal ganglia, the thalamus and the cerebellum in both schizophrenia and OCD [49] and have identified dissociated prefrontal cortex connection with the basal ganglia in both conditions [51, 52]. In more detail, evidence suggests a particular “prefrontal” dysfunction in schizophrenia and “orbitofrontal” dysfunctions in OCD patients [53].

The heterogeneous clinical presentation of comorbid OCS with symptom changes in the majority of our sample suggests more than a shared genetic and/or neurobiological vulnerability. The observed association between symptom remission might reflect an interaction between symptom clusters, which functionally influence each other [54]. Another possibility is that the course of symptoms over time is influenced by individual and environmental factors. Increased sensitivity to stress, negative affectivity and dysfunctional coping have, for example, been shown to constitute key components underlying both symptoms of OCD [55, 56] and psychosis [57, 58]. Recent investigations suggest that these mechanisms might also be crucial determinants of their co-occurrence [7, 59]. As briefly mentioned above, pre-existing higher emotional distress in the two de novo groups could possibly mirror heightened levels of anxiety and stress sensitivity.

Furthermore, the development of de novo OCS under antipsychotic medication, especially clozapine, has been increasingly acknowledged [60, 61]. Considering this effect within our data, more patients in the de novo and persistent groups were treated with clozapine when compared to individuals in the no-OCS group. Because clozapine is the treatment of choice in treatment-resistant symptoms, it might also explain why patients in the de novo group reported higher severity of psychotic symptoms before OCS onset.

Due to the observational nature of our study, the discussion of possible mechanisms remains speculative. We are well aware that assessing OCS only twice over the 3-year period is a main limitation of our study and precludes answering questions of causality. To further unravel the time course and possible co-variation between changes in psychotic, affective and obsessive–compulsive symptoms, and investigate underlying effect of individual and environmental factors such as stress sensitivity, coping skills and medication, prospective investigations with more frequent assessments are needed.

We acknowledge further limitations: We tried to account for relevant differences in age of onset and illness duration between our groups by including these variables as covariates in our analyses. However, we cannot completely rule out that higher severity and persistence of psychotic symptoms in our persistent OCS group is partly due to a more chronic and severe course of schizophrenia, independent of co-occurring OCS. Furthermore, because reliable information on the current intake of antipsychotic and antidepressant medication was only available in a subsample of our study, we did not account for cross-sectional between-group differences in our analyses. However, we did not find differences in treatment or illness duration between the two groups reporting changes in OCS, and significant associations between the presence of OCS and severity of subclinical psychotic symptoms in siblings strongly suggest that the link between the two conditions is more than just a medication effect. We further acknowledge that the mean severity of OCS in our groups was relatively low due to the fact that patients with relevant, although mild OCS were included in the OCS groups. Hence, compared to patient groups with more severe OCS, our results might underestimate associations with other clinical variables and the level of functioning.

In conclusion, our results suggest that the presence of at least mild co-occurring OCS is associated with greater severity of psychotic and affective symptoms and indicates lower levels of overall social and vocational functioning and additional burden for the affected patients, especially when OCS persist over time. The remission or absence of OCS on the other hand is associated with an improvement in psychotic and affective symptoms, respectively, and strengthens the need for a better understanding of the co-occurrence of OCS with other symptoms and clinical research aimed to develop and test treatment options. Comparable associations in a sample of non-psychotic drug naive siblings support the view that associations between OCS and symptom dimensions of schizophrenia are at least partly unrelated to treatment effects.

## Electronic supplementary material

Below is the link to the electronic supplementary material.

Supplementary material 1 (DOC 34 kb)

## Abbreviations

CAPE: Community Assessment of Psychic Experiences
GAF: General Assessment of Functioning
OCS: Obsessive–compulsive symptoms
PANSS: Positive and Negative Syndrome Scale

## References

[1] Swets M, Dekker J, van Emmerik-van Oortmerssen K, Smid GE, Smit F, de Haan L, Schoevers RA (2014). The obsessive compulsive spectrum in schizophrenia, a meta-analysis and meta-regression exploring prevalence rates. Schizophr Res.

[2] Achim AM, Maziade M, Raymond E, Olivier D, Merette C, Roy MA (2011). How prevalent are anxiety disorders in schizophrenia? A meta-analysis and critical review on a significant association. Schizophr Bull.

[3] Buckley P, Hwang M, De Haan L, Schirmbeck F, Zink M (2015). Comorbid psychiatric disorders in schizophrenia: more than just a chance co-occurrence. Obsessive–compulsive symptoms in schizophrenia.

[4] Cederlof M, Lichtenstein P, Larsson H, Boman M, Ruck C, Landen M, Mataix-Cols D (2015). Obsessive–compulsive disorder, psychosis, and bipolarity: a longitudinal cohort and multigenerational family study. Schizophr Bull.

[5] Dowling FG, Pato MT, Pato CN (1995). Comorbidity of obsessive–compulsive and psychotic symptoms: a review. Harv Rev Psychiatry.

[6] Stengel E (1945). A study on some clinical aspects of the relationship between obsessional neurosis and psychotic reaction types. J Ment Sci.

[7] Lysaker PH, Whitney KA, Davis LW (2006). Obsessive–compulsive and negative symptoms in schizophrenia: associations with coping preference and hope. Psychiatry Res.

[8] de Haan L, Sterk B, van der Valk R (2013). Presence of obsessive compulsive symptoms in first-episode schizophrenia or related disorders is associated with subjective well-being and quality of life. Early Interv Psychiatry.

[9] Fenton WS, McGlashan TH (1986). The prognostic significance of obsessive–compulsive symptoms in schizophrenia. Am J Psychiatry.

[10] de Haan L, Sterk B, Wouters L, Linszen DH (2012). The 5-year course of obsessive–compulsive symptoms and obsessive–compulsive disorder in first-episode schizophrenia and related disorders. Schizophr Bull.

[11] Schirmbeck F, Rausch F, Englisch S, Eifler S, Esslinger C, Meyer-Lindenberg A, Zink M (2012). Stable cognitive deficits in schizophrenia patients with comorbid obsessive–compulsive symptoms: a 12 months longitudinal study. Schizophr Bull.

[12] Varlakova Y, Patel D, Mukhopadhaya K, Laws K, David E, Sukwinder K, Fineberg N, De Haan L, Schirmbeck F, Zink M (2015). The neurocognitive and behavioural impact of comorbid obsessive–compulsive syndrome in schizophrenia. Obsessive–compulsive symptoms in schizophrenia.

[13] Hwang MY, Kim SW, Yum SY, Opler LA (2009). Management of schizophrenia with obsessive–compulsive features. Psychiatr Clin North Am.

[14] Cunill R, Castells X, Simeon D (2009). Relationships between obsessive–compulsive symptomatology and severity of psychosis in schizophrenia: a systematic review and meta-analysis. J Clin Psychiatry.

[15] Lysaker PH, Lancaster RS, Nees MA, Davis LW (2004). Patterns of obsessive–compulsive symptoms and social function in schizophrenia. Psychiatry Res.

[16] Öngür D, Goff DC (2005). Obsessive–compulsive symptoms in schizophrenia: associated clinical features, cognitive function and medication status. Schizophr Res.

[17] Guillem F, Satterthwaite J, Pampoulova T, Stip E (2009). Relationship between psychotic and obsessive compulsive symptoms in schizophrenia. Schizophr Res.

[18] Hunter H, Lysaker P, De Haan L, Schirmbeck F, Zink M (2015). Associations of comorbid obsessive–compulsive symptoms with psychotic and affective symptoms and general functioning. Obsessive–compulsive symptoms in schizophrenia.

[19] Nasrollahi N, Bigdelli I, Mohammadi MR, Hosseini SM (2012). The relationship between obsessions and compulsions and negative and positive symptoms in schizophrenia. Iran J Psychiatry.

[20] Poyurovsky M, Faragian S, Shabeta A, Kosov A (2008). Comparison of clinical characteristics, co-morbidity and pharmacotherapy in adolescent schizophrenia patients with and without obsessive–compulsive disorder. Psychiatry Res.

[21] Faragian S, Pashinian A, Fuchs C, Poyurovsky M (2009). Obsessive–compulsive symptom dimensions in schizophrenia patients with comorbid obsessive–compulsive disorder. Prog Neuropsychopharmacol Biol Psychiatry.

[22] Tibbo P, Kroetsch M, Chue P, Warneke L (2000). Obsessive–compulsive disorder in schizophrenia. J Psychiatr Res.

[23] Poyurovsky M, Fuchs C, Weizman A (1999). Obsessive–compulsive disorder in patients with first-episode schizophrenia. Am J Psychiatry.

[24] de Haan L, Hoogenboom B, Beuk N, van Amelsvoort T, Linszen D (2005). Obsessive–compulsive symptoms and positive, negative, and depressive symptoms in patients with recent-onset schizophrenic disorders. Can J Psychiatry.

[25] Bottas A, Cooke RG, Richter MA (2005). Comorbidity and pathophysiology of obsessive–compulsive disorder in schizophrenia: is there evidence for a schizo-obsessive subtype of schizophrenia?. J Psychiatry Neurosci.

[26] Lysaker PH, Whitney KA (2009). Obsessive–compulsive symptoms in schizophrenia: prevalence, correlates and treatment. Expert Rev Neurother.

[27] Kim SW, Jeong BO, Kim JM, Shin IS, Hwang MY, Amminger GP, Nelson B, Berk M, McGorry P, Yoon JS (2015). Associations of obsessive–compulsive symptoms with clinical and neurocognitive features in schizophrenia according to stage of illness. Psychiatry Res.

[28] Fontenelle LF, Lin A, Pantelis C, Wood SJ, Nelson B, Yung AR (2011). A longitudinal study of obsessive–compulsive disorder in individuals at ultra-high risk for psychosis. J Psychiatr Res.

[29] Korver N, Quee PJ, Boos HBM, Simons CJP, de Haan L, GROUP I (2012). Genetic Risk and Outcome of Psychosis (GROUP), a multi site longitudinal cohort study focused on gene-environment interaction: objectives, sample characteristics, recruitment and assessment methods. Int J Methods Psychiatr Res.

[30] American Psychiatric Association (2000) Diagnostic and statistical manual of mental disorders, 4th edn. Washington, DC.

[31] Andreasen NCFM (1992). The comprehensive assessment of symptoms and history (cash): an instrument for assessing diagnosis and psychopathology. Arch Gen Psychiatry.

[32] Wing JKBT, Brugha TT (1990). Scan: schedules fonr clinical assessment in neuropsychiatry. Arch Gen Psychiatry.

[33] Organisation WH (1992). The Composite International Diagnostic Interview (CIDI). Authorized core version 1.0.

[34] Goodman WK, Price LH, Rasmussen SA, Mazure C (1989). The Yale-Brown Obsessive Compulsive Scale: I. Development, use, and reliability. Arch Gen Psychiatry.

[35] de Haan L, Hoogeboom B, Beuk N, Wouters L, Dingemans PM, Linszen DH (2006). Reliability and validity of the Yale-Brown Obsessive–Compulsive Scale in schizophrenia patients. Psychopharmacol Bull.

[36] Boyette L, Swets M, Meijer C, Wouters L, Authors GROUP (2011). Factor structure of the Yale-Brown Obsessive–Compulsive Scale (Y-BOCS) in a large sample of patients with schizophrenia or related disorders and comorbid obsessive–compulsive symptoms. Psychiatry Res.

[37] Kay SR, Fiszbein A, Opler LA (1987). The positive and negative syndrome scale (PANSS) for schizophrenia. Schizophr Bull.

[38] van der Gaag M, Hoffman T, Remijsen M, Hijman R, de Haan L, van Meijel B, van Harten PN, Valmaggia L, de Hert M, Cuijpers A, Wiersma D (2006). The five-factor model of the Positive and Negative Syndrome Scale II: a ten-fold cross-validation of a revised model. Schizophr Res.

[39] Stefanis NC, Hanssen M, Smirnis NK, Avramopoulos DA, Evdokimidis IK, Stefanis CN, Verdoux H, van Os J (2002). Evidence that three dimensions of psychosis have a distribution in the general population. Psychol Med.

[40] Jones SH, Thornicroft G, Coffey M, Dunn G (1995). A brief mental health outcome scale-reliability and validity of the Global Assessment of Functioning (GAF). Br J Psychiatry.

[41] Pedersen G, Karterud S (2012). The symptom and function dimensions of the Global Assessment of Functioning (GAF) scale. Compr Psychiatry.

[42] Bosanac P, Mancuso SG, Castle DJ (2016). Anxiety symptoms in psychotic disorders: results from the Second Australian National Mental Health Survey. Clin Schizophr Relat Psychoses.

[43] Murphy DL, Timpano KR, Wheaton MG, Greenberg BD, Miguel EC (2010). Obsessive–compulsive disorder and its related disorders: a reappraisal of obsessive–compulsive spectrum concepts. Dialogues Clin Neurosci.

[44] Rickelt J, Viechtbauer W, Lieverse R, Overbeek T, van Balkom AJ, van Oppen P, van den Heuvel OA, Marcelis M, Eikelenboom M, Tibi L, Schruers KR (2016) The relation between depressive and obsessive–compulsive symptoms in obsessive–compulsive disorder: results from a large, naturalistic follow-up study. J Affect Disord 203:241–247 10.1016/j.jad.2016.06.009 27310102

[45] Schirmbeck F, Zink M (2013). Cognitive behavioural therapy for obsessive–compulsive symptoms in schizophrenia. Cogn Behav Therapist.

[46] de Haan L, Zink M, De Haan L, Schirmbeck F, Zink M (2015). Clinical presentation of obsessive–compulsive symptoms in patients with psychotic disorders psychopathological concepts, differential diagnosis, and symptom presentation. Obsessive–compulsive symptoms in schizophrenia.

[47] Schirmbeck F, Swets M, de Haan L, De Haan L, Schirmbeck F, Zink M (2015). Epidemiology: prevalence and clinical characteristics of obsessive–compulsive disorder and obsessive–compulsive symptoms in patients with psychotic disorders. Obsessive–compulsive symptoms in schizophrenia.

[48] Poyurovsky M, Kriss V, Weisman G, Faragian S, Schneidman M, Fuchs C, Weizman A, Weizman R (2005). Familial aggregation of schizophrenia-spectrum disorders and obsessive–compulsive associated disorders in schizophrenia probands with and without OCD. Am J Med Genet Part B Neuropsychiatr Genet.

[49] Rao NP, Venkatasubramanian G, De Haan L, Schirmbeck F, Zink M (2015). Obsessive–compulsive symptoms in schizophrenia: neurophysiological and neuroimaging findings. Obsessive–compulsive symptoms in schizophrenia.

[50] Scotti-Muzzi E, Saide OL (2016) Schizo-obsessive spectrum disorders: an update. CNS Spectr 27:1–15 10.1017/S1092852916000390 27669819

[51] Saxena S, Brody AL, Schwartz JM, Baxter LR (1998). Neuroimaging and frontal-subcortical circuitry in obsessive–compulsive disorder. Br J Psychiatry.

[52] Weinberger DR, Egan MF, Bertolino A, Callicott JH, Mattay VS, Lipska BK, Berman KF, Goldberg TE (2001). Prefrontal neurons and the genetics of schizophrenia. Biol Psychiatry.

[53] Cavallaro R, Cavedini P, Mistretta P, Bassi T, Angelone SM, Ubbiali A, Bellodi L (2003). Basal-corticofrontal circuits in schizophrenia and obsessive–compulsive disorder: a controlled, double dissociation study. Biol Psychiatry.

[54] Lysaker PH, Marks KA, Picone JB, Rollins AL, Fastenau PS, Bond GR (2000). Obsessive and compulsive symptoms in schizophrenia: clinical and neurocognitive correlates. J Nerv Ment Dis.

[55] Robinson LJ, Freeston MH (2014). Emotion and internal experience in obsessive compulsive disorder: reviewing the role of alexithymia, anxiety sensitivity and distress tolerance. Clin Psychol Rev.

[56] Cougle JR, Timpano KR, Sarawgi S, Smith CM, Fitch KE (2013). A multi-modal investigation of the roles of distress tolerance and emotional reactivity in obsessive–compulsive symptoms. Anxiety Stress Coping.

[57] Myin-Germeys I, van Os J, Schwartz JE, Stone AA, Delespaul PA (2001). Emotional reactivity to daily life stress in psychosis. Arch Gen Psychiatry.

[58] Nugent KL, Chiappelli J, Rowland LM, Daughters SB, Hong LE (2014). Distress intolerance and clinical functioning in persons with schizophrenia. Psychiatry Res.

[59] Schirmbeck F, Boyette L-L, Rvd Valk, Meijer C, Dingemans P, Van R, de Haan L, Kahn RS, de Haan L, van Os J, Wiersma D, Bruggeman R, Cahn W, Meijer C, Myin-Germeys I (2015). Relevance of five-factor model personality traits for obsessive–compulsive symptoms in patients with psychotic disorders and their un-affected siblings. Psychiatry Res.

[60] Schirmbeck F, Esslinger C, Rausch F, Englisch S, Meyer-Lindenberg A, Zink M (2011). Antiserotonergic antipsychotics are associated with obsessive–compulsive symptoms in schizophrenia. Psychol Med.

[61] Schirmbeck F, Zink M, De Haan L, Schirmbeck F, Zink M (2015). Effects of antipsychotic treatment on obsessive–compulsive symptoms. Obsessive–compulsive symptoms in schizophrenia.

