# Supplementary material for: Obsessive–compulsive symptoms and overall psychopathology in psychotic disorders: longitudinal assessment of patients and siblings
Source: Eur Arch Psychiatry Clin Neurosci. 2016 Dec 17;268(3):279–89. doi: 10.1007/s00406-016-0751-0 (PMC5861177; doi:10.1007/s00406-016-0751-0)
Supplement: Supplementary file 1 — Supplementary material 1 (DOC 34 kb) [file 406_2016_751_MOESM1_ESM.doc]

**Table 1 supplementary material: Relative risk of OCS occurrence in siblings based on their sibling(patient) group compared to the no-OCS sibling(patient) group**

|  | **OCS in siblings at baseline** | | **Relative risk compared to the No-OCS group** |
| --- | --- | --- | --- |
|  | **No-OCS**  **N (%)** | **OCS N (%)** | **RR (95% CI)** |
| **Patient group:** No OCS | 303 (95.4) | 15 (4.7) |  |
| Initial OCS | 59 (95.2) | 3 (4.8) | 1.03 (0.31-3.44) |
| Persistent OCS | 30 (93.8) | 2 (6.2) | 1.33 (0.32-5.54) |
| *De novo* OCS | 52 (96.4) | 3 (5.4) | 1.16 (0.35- 3.86) |
|  | **Follow-up** | |  |
|  | **No-OCS**  **N (%)** | **OCS**  **N (%)** |  |
| No OCS | 306 (96.3) | 12 (3.7) |  |
| Initial OCS | 59 (95.2) | 3 (4.8) | 1.28 (0.37- 4.41) |
| Persistent OCS | 30 (93.8) | 2 (6.2) | 1.66 (0.39-7.08) |
| *De novo* OCS | 53 (96.4) | 2 (3.6) | 0.96 (0.22- 4.19) |

Legend: CI: Confidence Interval; OCS: Obsessive-Compulsive Symptoms; RR: relative risk
